# Localization Algorithm for 3D Sensor Networks: A Recursive Data Fusion Approach

**DOI:** 10.3390/s21227626

**Published:** 2021-11-17

**Authors:** Rafaela Villalpando-Hernandez, Cesar Vargas-Rosales, David Munoz-Rodriguez

**Affiliations:** 1 School of Engineering and Sciences, Tecnologico de Monterrey, Monterrey C.P. 64849, Mexico; cvargas@tec.mx; 2 Aerospace Development Center, Instituto Politecnico Nacional, Ciudad de Mexico C.P. 06000, Mexico; dmunoz@itesm.mx

**Keywords:** recursive localization, position information fusion, 3D sensor networks

## Abstract

Location-based applications for security and assisted living, such as human location tracking, pet tracking and others, have increased considerably in the last few years, enabled by the fast growth of sensor networks. Sensor location information is essential for several network protocols and applications such as routing and energy harvesting, among others. Therefore, there is a need for developing new alternative localization algorithms suitable for rough, changing environments. In this paper, we formulate the Recursive Localization (RL) algorithm, based on the recursive coordinate data fusion using at least three anchor nodes (ANs), combined with a multiplane location estimation, suitable for 3D ad hoc environments. The novelty of the proposed algorithm is the recursive fusion technique to obtain a reliable location estimation of a node by combining noisy information from several nodes. The feasibility of the RL algorithm under several network environments was examined through analytic formulation and simulation processes. The proposed algorithm improved the location accuracy for all the scenarios analyzed. Comparing with other 3D range-based positioning algorithms, we observe that the proposed RL algorithm presents several advantages, such as a smaller number of required ANs and a better position accuracy for the worst cases analyzed. On the other hand, compared to other 3D range-free positioning algorithms, we can see an improvement by around 15.6% in terms of positioning accuracy.

## 1. Introduction

New paradigms such as the Internet of things, Industry 4.0, health monitoring, assisted living and physical and environmental conditions monitoring, among other applications, have been introduced due to the increased reliability, improved autonomy and low-cost trend of sensor networks which enable the market growth for both military and civil applications. Data fusion techniques can be applied to find solutions in several challenges that must be addressed when designing new applications or techniques for sensor networks. Connectivity and network topology in ad hoc and sensor networks change dynamically due to variations in the propagation conditions and mobility, [1]. At the same time, in nowadays network scenarios, position location awareness becomes more relevant in order to achieve network goals such as fast routing, efficient resource management, sensor energy harvesting and reliable deployment of location-aware services. Besides GPS and other GNSS systems meant to provide three-dimensional geolocation for outdoor applications, most existing positioning techniques deal with planar location environments that are not sufficiently suitable for many applications. Although GPS has been in place for many years for several uses, its availability and accuracy are insufficient for indoor applications and other compromised scenarios such as tunnels, mines and high-vegetation environments. Furthermore, in sensor networks, range-based location techniques might not be reliable due to range-noisy estimations obtained from complex physical environments. In real scenarios, sensors might fail, burn power batteries or be under different sources of interference; so, it is required to design alternative estimation methods to address these random restrictions.

Therefore, data fusion-based techniques represent a crucial alternative for multiple network applications, such as localization. Note that, in sensor positioning, data fusion can be understood as the set of techniques or methods that process or combine data from several sensors in order to obtain a better location estimation of a node of interest [2,3]. Therefore, alternative 3D localization schemes are necessary for applications such as emergency call services in urban and metropolitan environments, museum guides, warehouse stock management, industrial plant monitoring and assisted living, among many others [4].

In the literature, we can find range-based and range-free sensor localization techniques, most of them designed for 2D scenarios. Basically, range-based localization techniques are focused on the improvement of the estimated distances (through TOA, DTOA and RSSI) between anchor nodes (ANs) and the node to locate or node of interest, [5]. Then, available ANs perform classical trilateration and the centroid of the intersected areas is selected as the final location estimation of the node of interest. Some of these proposals use Kalman filters and/or modifications of the Kalman filter in order to improve estimated distances [6,7,8] and thus the location estimation. Others have focused on the improvement of the centroid calculation of intersected areas, while some authors have proposed some variations such as the weighted centroid calculation [9].

One of the first range-based 3D localization techniques was proposed in [6], where they introduced a method based on two stages. During the first stage, nodes with known locations estimate distances based on the received signal strength indicator (RSSI) to a mobile location-assistant (LA) such as an aircraft, a balloon, a robot, a vehicle, etc. Then, each location-unaware sensor discovers its position by observing the moving, location-aware LA nodes. In the second stage, the location-unaware sensor nodes use an unscented Kalman filter for self-localization. This technique reports the requirement of a high number of known location anchor nodes in order to achieve moderate position errors. For instance, it is reported that, in low complexity scenarios with 200 sensor nodes deployed in a 1000 × 1000 × 100 feet space, they require 5% of anchor nodes in order to achieve position errors of around 11 feet for flat terrains. As they evaluate more complex scenarios, the accuracy is degraded to positioning errors of about 100 feet. Although this method does not rely on network density and topology, its performance depends on the observation of mobile devices that might not be available in some hostile scenarios; additionally, its performance degrades as the terrain complexity increases.

Another popular range-based approach was introduced in [7] for sensor node localization in planar scenarios, using a multidimensional scaling (MDS) technique. An extrapolated concept for 3D scenarios is presented in [10], where the authors propose an MDS-MAP-based localization technique where they calculate the shortest distances between every pair of nodes, then they apply classical multidimensional scaling to the distance matrix. Finally, the relative map with relative location for each node is obtained. In order to transform the relative map into a global map, at least four anchor nodes are needed. The main limitation of this algorithm is a strong dependency on the input information for the distance matrix and the high dependency on the network connectivity. The reported performance of the proposed algorithm considers a scenario with 100 nodes in a 100*r* × 100*r* × 100*r* (*r* is the unit length distance) space, varying connectivity. The position error reported is of about 12%*R* (*R* is the maximum transmission range) with high connectivity and a high number of anchors (10 anchors).

For comparison purposes, we can observe that the two algorithms just discussed in the previous paragraphs require at least 10 ANs to be implemented, while our proposed algorithm can be implemented using only three ANs. In terms of accuracy, the purpose of this paper is not to find a direct comparison between the worst analyzed cases. However, we achieve position errors of around 10%*r*_max_ (*r*_max_ is the maximum transmission range) using only three ANs, while, in [6], the authors achieve an accuracy of around 45%*r*_max_ and, in [7], the authors achieve 12%*r*_max_ for the worst analyzed cases.

In [11], the authors present a range-based (RSSI estimations) sequence localization algorithm where they construct a 3D Voronoi diagram, which is used to divide the operational scenario depending on known location nodes. Then, they formulate a rank sequence table of estimated location nodes (virtual beacons) based on the previous partition. RSSI estimations between nodes are used to correct the measured distances and to fix the location sequence of unknown nodes. The location error is normalized to the transmission range *r*_0_, achieving a location error of around 0.12*r*_0_ for eight ANs.

In range-free localization methods, the distance between ANs and the node of interest is based on the hop count criterion; therefore, the position estimation of the node does not depend on the accuracy of the estimated distances between anchor nodes and the node to locate. Most of the range-free localization methods base their approaches on the assumption that the Euclidean distance between nodes is proportional to the number of hops in the connecting route. Then, a heuristic method to estimate the coordinates of the node of interest is applied. In [12], the authors propose a range-free approach, where a position location (PL) method suitable for 3D environments is introduced. This method is based on the convenient deployment of access points (APs) or anchor nodes and on the discretization of the network space. They base distance estimation on the hop count criterion on a Manhattan scenario, [13]. A set of algebraic relations is defined for the node’s coordinates based on the distances from the APs to the node of interest. In addition, a maximum a posteriori probability (MAP) criterion is used to improve the estimated distances and performance of the algebraic method. They deploy from 300 to 600 nodes in a 10*l* × 10*l* × 10*l* space (*l* = Manhattan step) and achieve localization errors of about 0.83*r*_max_ (where *r*_max_ is the maximum transmission range), which can be considered a good result for the free range/multihop scenarios analyzed.

Comparing the proposed method against the last 3D positioning methods summarized, we achieve an improvement of around 15.6% for similar scenarios, using 4 ANs, 600 deployed nodes and hop count as distance estimation criterion.

In this paper, we propose a novel localization technique named Recursive Localization (RL) algorithm, suitable for 3D sensor node positioning. In the proposed RL algorithm, the initial node of interest’s coordinates are estimated by classical trilateration techniques, after a reference plane is constructed based on the locations of only three available ANs. Then, the recently located node ***n_j_*** becomes a new anchor node and one of the original ANs is now considered to be a node of interest to be located. Since the actual locations of the ANs are known, we obtain the difference between the AN’s estimated location using ***n_j_*** and the AN’s true location by least squares. Finally, we use this difference (called recursive correcting vector, or **CV**) to recalculate the location of the node of interest ***n_j_***. The algorithm is explained in detail in the following sections. Note that, when more than three ANs are available, we can take advantage of having several reference planes to improve the first location estimation of ***n_j_*** (this is the starting point of the RL). Note that observations are prone to impairments due to either the error in distance estimations, anchor node movements or the absence of direct connectivity from sensor nodes to ANs, rendering in ad hoc routes that may depart from a line of sight (LOS) trajectory. The performance of the proposed methodology is assessed for both LOS and multihop scenarios. Different scenarios are analyzed, where several network factors are modified, such as the number of ANs, their relative positions, the number of possible reference planes and the maximal transmission range, as well as the node densities for multi-hop scenarios. The performance in a noisy environment is considered. The proposed RL algorithm provides a significant improvement of the location accuracy for all the scenarios analyzed, without the need of additional information or processing for distance estimation improvement with a very low number of ANs.

In Table 1, we summarize the characteristics of the main positioning methods described above in order to clarify the differences between the state of the art and the proposed method. We see further that the proposed RL algorithm can be used under both the range-based or free-range-based approach.

In [14], the authors formulate a taxonomy for localization algorithms’ classification. They also present a study of different main localization algorithms along with their improved versions. The challenges and research approaches of localization in sensor networks emerging applications are also discussed.

In Section 2, we introduce the scenario used for position location with the description of the Recursive Localization algorithm. Section 3 contains the simulation results showing the performance as several parameters are varied and, in Section 4, the conclusions are discussed.

## 2. Positioning System

**Figure 1:**
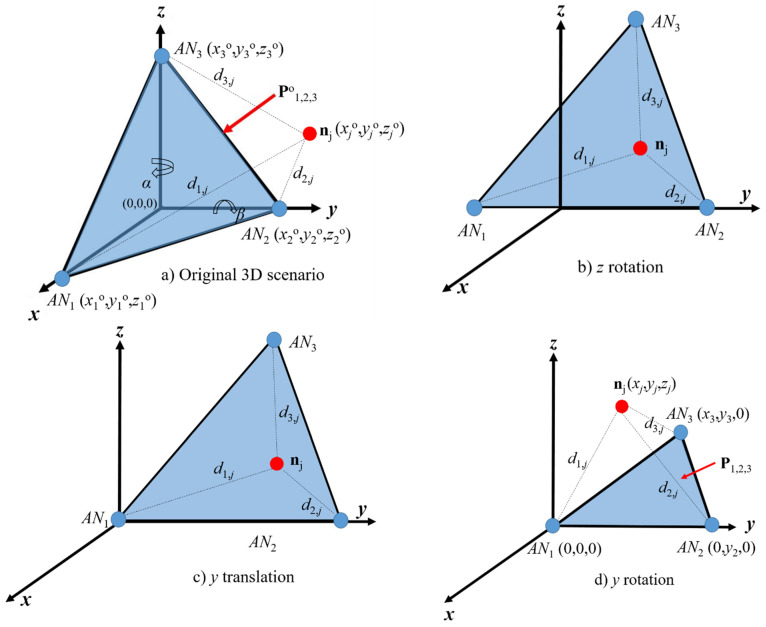
(**a**) Original 3D network scenario with three ANs. (**b**) Rotated reference plane with respect to the *z*-axis by angle *α*. (**c**) Translated reference plane. (**d**) Rotated reference plane with respect to the *y*-axis by angle *β*.

In a basic network set up, we want to determine the location of a node in a defined 3D scenario. The node is considered to be able to establish direct or multihop routes to several ANs that provide global connectivity. The reachability of the node of interest to at least three anchor nodes, *AN*_1_, *AN*_2_ and *AN*_3_, with corresponding known coordinates (*x_k_*, *y_k_*, *z_k_*), *k =* 1, 2, 3, is assumed. Note that the node to be located can be a radio source associated to a sensor with initial unknown location; this is a feasible scenario, as, in some cases, sensors may be deployed without precise location information. Anchor nodes are notified to perform distance estimations to the node of interest. This information is sent to the node so that it can estimate its own location. Any of three ANs (*AN_k_*, *AN_l_*, or *AN_n_*) with original coordinates (*x_k_*^o^, *y_k_*^o^, *z_k_*^o^), (*x_l_*^o^, *y_l_*^o^, *z_l_*^o^) and (*x_n_*^o^, *y_n_*^o^, *z_n_*^o^), respectively, can be selected to define the original reference plane **P**^o^*_kln_* so that coordinates (*x_j_*^o^, *y_j_*^o^, *z_j_*^o^) of the node to be located ***n****_j_* can be expressed with respect to the reference plane. For instance, see the plane **P**^o^_123_ defined by *AN*_1_, *AN*_2_ and *AN*_3_ in Figure 1a forming a reference for the location of node ***n****_j_*.

As illustrated in Figure 1, the location estimation of the node of interest, calculated by anchor nodes that define the reference plane (Figure 1a), is relative to the rotated/translated anchor’s coordinates (Figure 1d). Note that, after rotation/translation, the coordinates of node *AN*_1_ are (0, 0, 0); therefore, in order to obtain the scenario illustrated in Figure 1b,c, a rotation by angles *α* and *β* is performed in the *z*- and *y*-axis, respectively. Therefore, the coordinates (*x_j_*, *y_j_*, *z_j_*) of node ***n****_j_* with respect to plane **P**_123_ can be obtained from the following distance relationships:

(1a) $$d_{1,j}^{2}=x_{j}^{2}+y_{j}^{2}+z_{j}^{2},$$

(1b) $$d_{2,j}^{2}={\left(x_{2}-x_{j}\right)}^{2}+y_{j}^{2}+z_{j}^{2},$$

(1c) $$d_{3,j}^{2}={\left(x_{3}-x_{j}\right)}^{2}+{\left(y_{3}-y_{j}\right)}^{2}+z_{j}^{2},$$

where we denote the true separation distance between node ***n****_j_* and *AN_k_* as *d*_*k*,*j*_ and this is unaltered after plane rotation/translation. Thus, after the algebraic handling and in a noise free scenario, the coordinates (*x_j_*, *y_j_*, *z_j_*) can be expressed as

(2a) $$x_{j}=\frac{d_{1,j}^{2}-d_{2,j}^{2}+x_{2}^{2}}{2x_{2}},$$

(2b) $$y_{j}=\frac{d_{1,j}^{2}-d_{3,j}^{2}+x_{3}^{2}+y_{3}^{2}-2x_{3}x_{j}}{2y_{3}},$$

(2c) $$z_{j}=\sqrt{d_{1,j}^{2}-{\left(x_{j}\right)}^{2}-{\left(y_{j}\right)}^{2}}\cdot$$

For the scenario illustrated in Figure 1, the coordinates (*x_j_*, *y_j_*, *z_j_*) can be translated back to the original coordinate system (*x_j_*^o^, *y_j_*^o^, *z_j_*^o^), so that node ***n****_j_* is meant (after the rotation in *y*- and *z*-axis) to be located at

(3) $$x_{j}^{o}=(x_{j}\cos\beta +z_{j}\sin\beta )\cos\alpha -y_{j}\sin\alpha ,\;x_{j}^{o}=(x_{j}\cos\beta +z_{j}\sin\beta )\cos\alpha -y_{j}\sin\alpha ,\;z_{j}^{o}=-x_{j}\sin\beta +z_{j}\cos\beta ,$$

where *α* and *β* are the rotation angles illustrated in Figure 1a. This process can be conducted for any other combination of ANs. Note that, in real environments, physical as well as propagation impairments render in noisy distance estimations from ANs to the node of interest. These impairments are quantified by using an additive noise model for estimation distances. Thus, let us define the estimated distance between *AN_k_* and the node of interest ***n****_j_* as *d*’_*k*,*j*_ = *d*_*k*,*j*_ + *E*_*k*,*j*_, where we assume that *E*_*k*,*j*_ is exponentially distributed with parameter *λ* (*E*_*k*,*j*_~exp(λ)), that is proportional to the true distance *d*_*k*,*j*_ from the *AN_k_* to node ***n****_j_*. With these distances estimated, the coordinates of the node of interest are not those obtained in a noise-free scenario and they are noisy coordinates (*x*’*_j_*, *y*’*_j_*, *z*’*_j_*) obtained by using the same equation set (2), but with distances given by *d*’*_k_*_,__*j*_, *k* = 1, 2, 3. In the following section, we introduce the RL algorithm, where the coordinates of the node of interest are recursively recalculated and where we express (*x*’*_l_^n^*, *y*’*_l_^n^*, *z*’*_l_^n^*) as the *n*-th calculation of the node of interest’s location coordinates. For the RL algorithm, the coordinates (*x*’*_j_*, *y*’*_j_*, *z*’*_j_*) obtained using Equation set (2) with *d*’*_k_*_,*j*_ are defined as (*x*’*_j_^n^*, *y*’*_j_^n^*, *z*’*_j_^n^*) for *n* = 1.

### 2.1. Recursive Localization (RL) Data Fusion Algorithm

**Figure 2:**
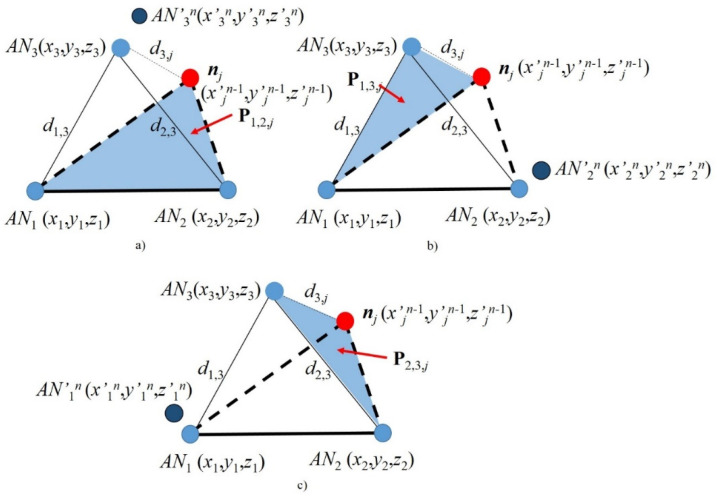
Recursive localization illustration. (**a**) *AN*_3_ self-locating, (**b**) *AN*_2_ self-locating and (**c**) *AN*_1_ self-locating.

As noted before, the estimated distances *d*’_*k*,*j*_ may be corrupted, resulting in impaired coordinate calculations. In this section, we explain the data fusion-based Recursive Localization (RL) algorithm. This algorithm is based on the ANs’ known locations and on the first location estimation (*x*’*_j_*^1^, *y*’*_j_*^1^, *z_j_*^1^) of the node of interest ***n****_j_*, as follows. Let us consider a scenario with three available ANs, *AN*_1_, *AN*_2_ and *AN*_3_, where we can estimate the location of a node of interest ***n****_j_*, (*x*’*_j_^n^*, *y*’*_j_^n^*, *z _j_^n^*), relative to plane **P***_klm_* (e.g., **P**_123_), based on a set of estimated distances *d*’_*k*,*j*_ (e.g., *k* = 1, 2, 3) as those in Equation set (2). Note that the super index of the coordinates (*x*’*_j_^n^*, *y*’*_j_^n^*, *z_j_^n^*) starts with *n* = 2 for the first time that the recursive algorithm recalculates the location of ***n****_j_* and it continues for values of *n* > 2 for the subsequent calculations. Once the coordinates (*x*’*_l_^n^*, *y*’*_l_^n^*, *z*’*_l_^n^*) are calculated, node ***n****_j_* becomes an anchor node *AN_j_* and three new different planes of reference, **P**_12*j*_, **P**_13*j*_ and **P**_23*j*_, can be generated by rotation, as shown in Figure 2. Note that, in each of these new planes, one of the original anchor nodes is disregarded, i.e., *AN*_3_ is not part of plane **P**_12*j*_; we call this the extra anchor node. Then, the RL data fusion algorithm is as follows:

1. The extra anchor node *AN_k_* (e.g., *AN*_3_ for reference plane **P**_12*j*_, see Figure 2a) performs self-localization (i.e., calculates the coordinates of *AN*’*_k_^n^*, namely, *x*’*_k_^n^*, *y*’*_k_^n^*, *z*’*_k_^n^*) based on the two noise-free distances with the other two original ANs (e.g., *d*_13_ and *d*_23_, if *AN*_3_ is self-locating) and one noisy distance (e.g., *d*’_3,*j*_, if *AN*_3_ is self-locating) with the estimated location of the new anchor node ***n****_j_*. For instance, if *AN*_3_ is self-locating, *x*_3_′*^n^*, *y*_3_′*^n^* and *z*_3_′*^n^* are calculated based on the distances *d*_1,3_, *d*_2,3_ and *d*’_3,*j*_ and on the previous found coordinates (*x*’*_j_^n^*^−1^, *y*’*_j_^n^*^−1^, *z*’_j_^*n*−1^), using Equation set (2). Let us recall that *n* − 1 = 1 for the first recursion.
2. The location error is defined as the Euclidean distance between the true position of *AN_k_* (*x_k_*, *y_k_*, *z_k_*) and the current *n*-th estimate of *AN*’*_k_^n^* (*x*’*_k_^n^*, *y*’*_k_^n^*, *z*’*_k_^n^*), i.e.,
  $${e_{k}}^{n}=\sqrt{{\left(x_{k}-x{’}_{k}{}^{n}\right)}^{2}+{\left(y_{k}-y{’}_{k}{}^{n}\right)}^{2}+{\left(z_{k}-z{’}_{k}{}^{n}\right)}^{2}}.$$

3. Then, we formulate the recursive correcting vector **CV***_k_*, which is used to re-calculate the node of interest coordinates, based on the difference between the estimated and the true position of *AN_k_*. This is calculated as **CV***_k_^n^* = [*cx_k_^n^* = |*x*’*_k_^n^* − *x_k_*|, *cy_k_^n^* = |*y*’*_k_^n^* − *y_k_*|, *cz_k_^n^* = |*z*’*_k_^n^* − *z_k_*|] (e.g., **CV**_3_*^n^* = [*cx*_3_*^n^* = |*x*’_3_*^n^* − *x*_3_|, *cy*_3_*^n^* = |*y*’_3_*^n^* − *y*_3_|, *cz*_3_*^n^* = |*z*’_3_*^n^* − *z*_3_|]).
4. Then, to find a local minimum of ***n****_j_* coordinates, we calculate a new estimate of the coordinates (*x*’*_j_^n^*, *y*’*_j_^n^*, *z*’*_j_^n^*) of node ***n****_j_* by applying **CV***_k_* as follows:
  *x*’*_j_^n^* = *x*’_*j*_^*n*−1^ − *cx_k_^n^*,
  *y*’*_j_^n^* = *y*’_*j*_^*n*−1^ − *cy_k_^n^*, (4)
  *z*’*_j_^n^* = *z*’_*j*_^*n*−1^ − *cz_k_^n^*,
  where *n* is the number of the current coordinate calculation in the RL algorithm.

5. Distances involved in equation set (2) are used again to re-calculate the location (*x*’*_k_^n^*, *y*’*_k_^n^*, *z*’*_k_^n^*) of anchor *AN*’*_k_^n^*; this process (steps 1–4) is repeated until a local minimum is found, that is, when the current error calculation *e_k_^n^* is larger than the previous error *e_k_^n−^*^1^. If this criterion is not met, the recursive algorithm continues until |*e_k_^n^* − *e_k_*_−1_*^n^*^−1^| < ξ, or when an unfeasible location is found, in order to proceed with the next step. Note that we are assuming that the node of interest is located at the first octant, as illustrated in Figure 1d.
6. Finally, the coordinates (*x*’*_j_^n^*, *y*’*_j_^n^*, *z*’*_j_^n^*) calculated in the last iteration are considered as the final location estimation of ***n****_j_*.

Please note that the convergence condition of the RL algorithm is defined in step 5. Simulations over different ξ in the range from 0.10*e_k_*_−1_*^n^*^−1^ to 0.30*e_k_*_−1_*^n^*^−1^ were performed and results showed a similar behavior.

In addition, note that the RL algorithm does not rely on the knowledge of the true position of ***n****_j_*_._ Let us recall that the local minimum as well as the threshold error ξ (step 5) are formulated based on successive calculations of the estimated locations of *AN_k_*. Note that the correcting factor is used to correct the position estimation of ***n****_j_* (step 4) and this new position is used to repeat steps 1–4 until the local minimum is found or the threshold error is reached (step 5).

In order to provide a better understanding of the complete localization process, we provide the following points of the execution of the algorithm:

- Calculate the initial location estimation (*x*’*_j_*^1^, *y*’*_j_*^1^, *z*’_j_^1^) of *n_j_* using Equation set (2).
- Then, node ***n****_j_* now becomes an anchor node.
- Next, obtain the reference planes **P**_12*j*_, **P**_13*j*_ and **P**_23*j*_ using the new anchor node ***n****_j_*.
- Then, follow steps 1–6 of the RL algorithm.

This algorithm can be implemented using a single extra anchor node AN or using the three ANs as extra anchor nodes and averaging the final ***n****_j_* location, which we call mean RL. We anticipate that using all the ANs involved might improve the ***n****_j_* location estimation.

Let us recall that the reference planes illustrated in Figure 2 are obtained by rotation and translation of the original plane by applying Equation set (3). Finally, when the location estimation of anchor *AN*’*_k_^n^* (*x*’*_k_^n^*, *y*’*_k_^n^*, *z*’*_k_^n^*) is obtained, it can be transformed by translation/rotation to the original reference plane in order to calculate the corresponding recursive **CV**.

### 2.2. Reference Plane Redundancy

**Figure 3:**
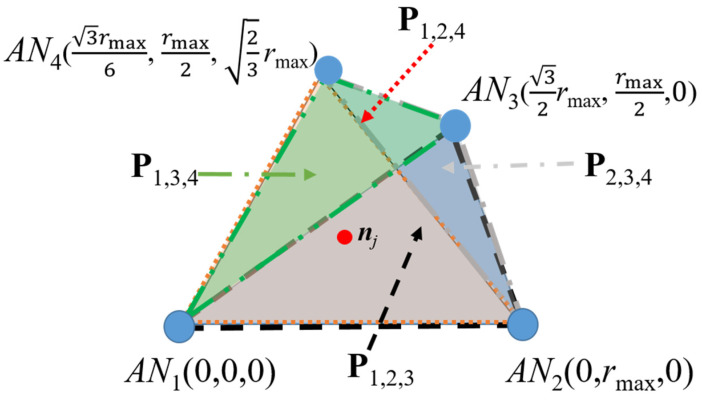
Different reference planes for several position location estimations of ***n****_j_* in a four-AN scenario.

Note that, if more ANs are available, then we can obtain alternative planes that can be used for the location estimation of ***n****_j_* and this may improve the first ***n****_j_* location estimation (*x*’*_j_*^1^,*y*’*_j_*^1^,*z*’*_j_*^1^) used in the first step of the RL algorithm. The number of different planes that we can select to be used in the algorithm is given by the number of combinations *_N_C*_3_, where *N* is the number of available ANs. Thus, for *N* = 4, four different sets of three ANs are obtained; therefore, four different calculations of coordinates (*x_j_*, *y_j_*, *z_j_*) are possible (see Figure 3). In a noise-free environment, these four sets of equations lead to the same location estimation result. However, in practice, the estimated distances *d*’*_k*,*j_* are obtained from field strength or delay measurements and are impaired by different propagation phenomena or routing strategies. All these impairments render in different coordinate calculations that can be averaged to smooth out the difference. In [15], the authors proposed a PL method for a different 2D scenario based on a set of equations similar to those in (1); however, they did not take advantage of anchor point redundancy as we do in this paper. They related the equations in (1) by subtracting them and arranging terms in a matrix form, solving for a 2D scenario.

## 3. Simulations and Results

Note that, for scenarios as those illustrated in Figure 1, the ANs’ locations can be arbitrarily set. In order to assess the performance of the proposed 3D PL technique and without loss of generality, we define the working operational space where three ANs are located at the corners of an equilateral triangle *AN*_1_ (0,0,0), *AN*_2_(*x*_2_ = *r*_max_,0,0) and *AN*_3_ (*x*_3_ = *r*_max_/2,0), where *r*_max_ is the maximal transmission range. For cases when four ANs are available, let us define a scenario such as that illustrated in Figure 3, where the ANs’ locations are placed at the corners of a regular tetrahedron. Therefore, for simulation purposes and without loss of generality, the ANs’ locations are set as *AN*_1_ (0,0,0), *AN*_2_ (0,*r*_max_,0), *AN*_3_ ($\sqrt{3}r_{\max}/2$,$\;r_{\max}/2$,0) and *AN*_4_ ($\sqrt{3}r_{\max}/6$,$\;r_{\max}/2$,$\;\sqrt{2}r_{\max}/3$).

Let us recall that we expect to have a distance estimation noise derived from signal propagation impairments. In the following, we present the results of the mean squared Euclidean distance error *ε* between the estimated and true locations, normalized to *r*_max_, i.e., *ε*/*r*_max_. The normalization allows us to observe the error as a proportion of the reachability radius and to see if, for a larger value of *r*_max_, this proportion does not change. This normalized location error is shown for an additive observation error *E_k_*_,*j*_ of the true separation between *AN_k_* and the node to be located, ***n****_j_*. Let us recall that *E*_*k*,*j*_ is exponentially distributed with parameter *λ* and proportional to the true distance *d*_*k*,*j*_ from *AN_k_* to node ***n****_j_*, *E*_*k*,*j*_~exp(*λ*) ∗ *d*_*k*,*j*_. This is the observed distance *d*’_*k*,*j*_ from *AN_k_* to ***n****_j_* and can be expressed as *d*’_*k*,*j*_ = *d*_*k*,*j*_ + *E*_*k*,*j*_. The simulations were conducted for a random location of node ***n****_j_* and the maximum coverage radius was initially set to *r*_max_ = 10 m.

**Figure 4:**
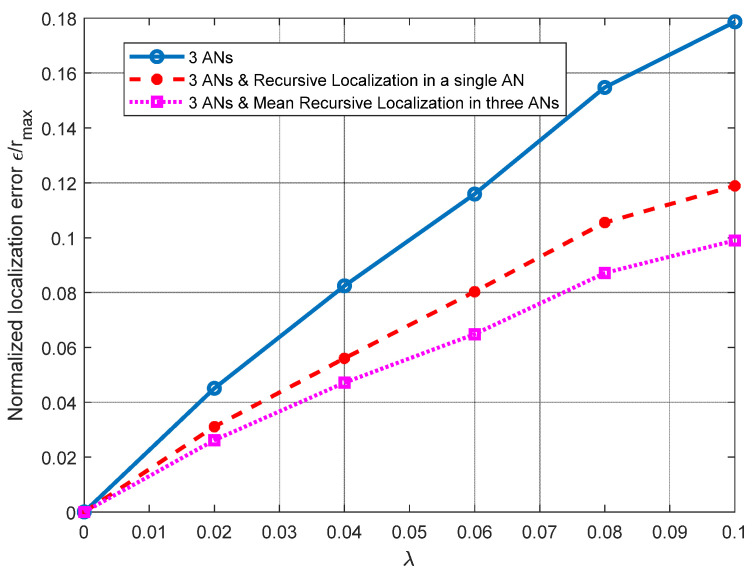
Normalized mean squared error *ε*/*r*_max_ vs. exponentially distributed with parameter *λ*, considering three ANs, single and mean RL for three ANs and *r*_max_ = 10 m.

For the results shown in Figure 4, a line of sight (LOS) from the ANs to node ***n****_j_* is considered, to observe how having noisy distance estimations may affect the performance of the PL 3D technique. In this figure, the results of the normalized Euclidean error *ɛ*/*r*_max_ are presented as a function of the parameter *λ* of the random variable—let us recall that *E*_*k*,*j*_~exp(*λ*) ∗ *d*_*k*,*j*_. Please note that both the mean and the variance of the exponential random variable of the error are related to *λ* (i.e., E[*E*_*k*,*j*_] = 1/*λ*, Var[*E*_*k*,*j*_] = 1/*λ*^2^). Please note that, as *λ* increases, so does the error dispersion. In the context of distance estimation, this means that we evaluate distance errors with high variability. We select *λ* from 0 to 0.1, to provide a high error dispersion. This simulation presents the results when using a single plane (three ANs available) and when single and mean RL are introduced on the node’s coordinates calculation. As noted in Figure 4, the RL algorithm achieves significant improvements, of about 30% and 40%, when using one AN and three ANs, respectively.

**Figure 5:**
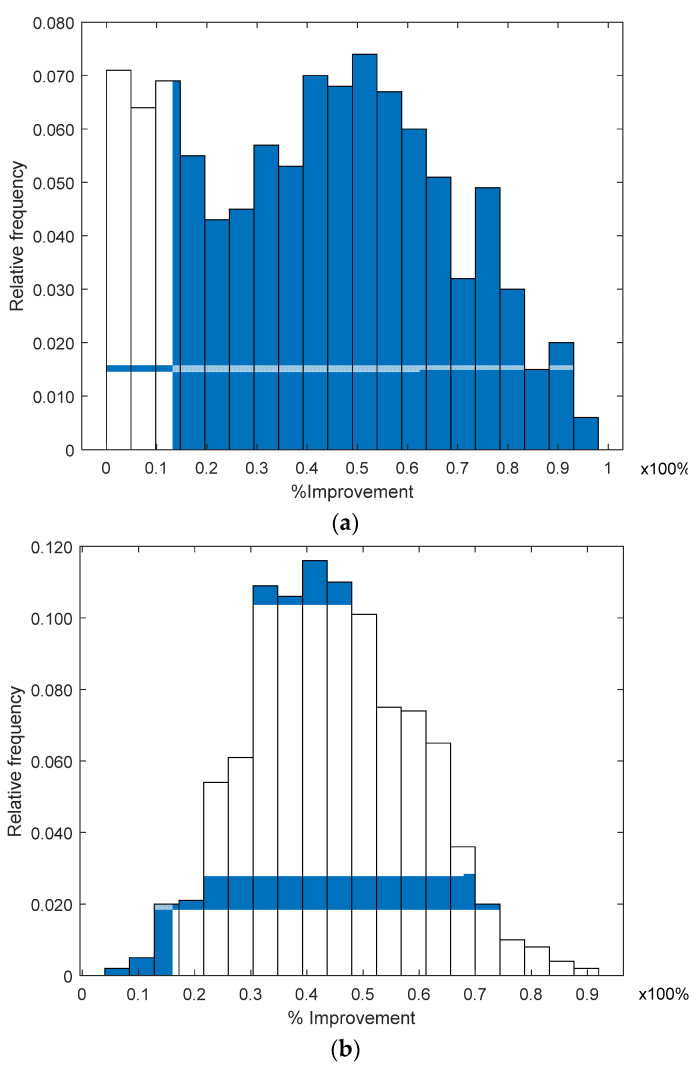
Improvement histogram achieved using RL: (**a**) single AN and (**b**) mean 3 ANs.

In Figure 5, we present a histogram of the percentage of improvement obtained with the RL algorithm over the location estimation using a single plane for *λ* = 0.1 and for 1000 analyzed cases. The improvement percentage *ρ* is defined as *ρ* = $\frac{\varepsilon -\varepsilon_{RL}}{\varepsilon }\;*100\%$, where ε is the Euclidean distance error using a single plane and $\varepsilon_{\mathrm{RL}}$ is the Euclidean distance error using the RL algorithm, between the estimated and the true node location. In Figure 5, we observe that the RL algorithm provides a significant improvement in all the analyzed cases, because all the values of *ρ* in Figure 5 are non-negative. The histograms show that the probability density function changes as more ANs are considered on the RL algorithm, concentrating improved values of *ρ* around the mean (see Figure 5b). Note that the improvement percentage *ρ* pdf for the mean RL (three involved ANs) is different from that of the *ρ* pdf using one AN. For the mean RL, *ρ* seems to have a normal pdf with a mean of around *ρ* = 50% and approximately 94.7% of the analyzed cases presented an improvement from *ρ* = 20% to *ρ =* 80%. When we take the average (mean RL) of the three ANs’ correction vectors, it is possible that the correction vector associated with a single AN is compensated by the other two estimations, providing a better behavior and avoiding any possible bias.

**Figure 6:**
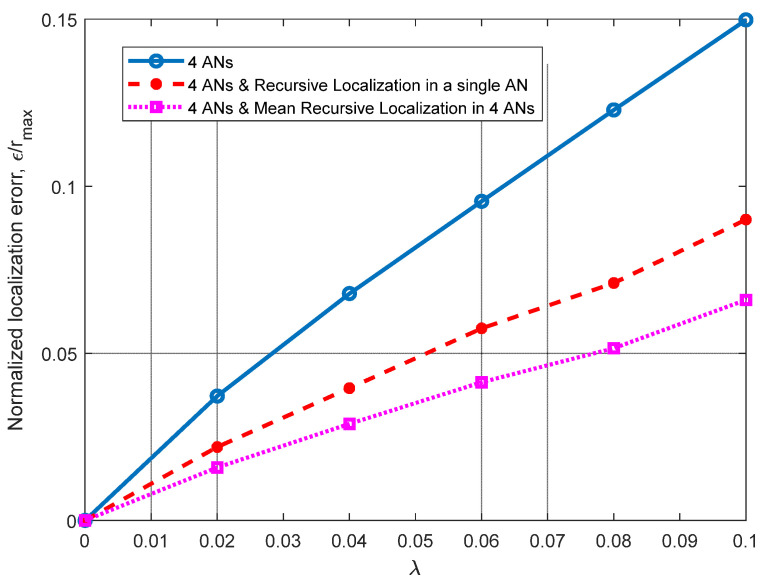
Normalized mean squared error *ε*/*r*_max_ vs. exponentially distributed distance error with parameter *λ*, considering four ANs, single and mean recursive localization and *r*_max_ = 10 m.

In Figure 6, additional planes are considered (four ANs) for (*x*’*_j_*, *y*’*_j_*, *z*’*_j_*) calculation and it can be observed that, by using additional planes, we achieve better results than those shown in Figure 4, where only a single plane (three ANs) is used. Note that we can perform recursive localization in a single AN or in the three ANs on the working plane and take the average of the three estimations (mean RL). This can be performed on a single plane or on several planes.

Moreover, it can be seen how the use of the RL algorithm in a single plane provides better results than using multiple reference planes alone. As it can be observed in Figure 4, the proposed RL method provides significant improvements in both scenarios. These improvements can also be increased when the mean RL is used in the four ANs involved—an improvement of about 26% over the results obtained using the RL algorithm on a single plane. As in the scenario analyzed in Figure 4, in this analysis, we can observe how the RL algorithm achieves significant improvements (single and mean) for all the cases analyzed (*σ*).

**Figure 7:**
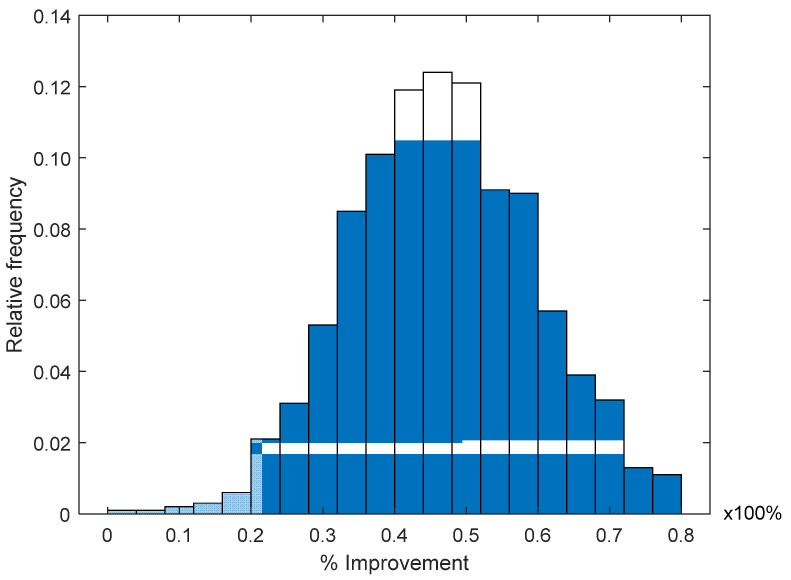
Improvement histogram for recursive localization using mean 4 ANs.

In Figure 7, we present the histogram of the improvement percentage *ρ*, obtained using the RL algorithm in the four ANs involved and averaging recursive vectors in order to obtain the final location estimation for ***n****_j_*. In this figure, we can observe that, as more ANs are considered in the RL algorithm, the pdf of the improvement *ρ* is more concentrated around the mean. For this evaluation, *ρ* seems to have a normal pdf with a mean of around *ρ* = 50% and approximately 99% of the analyzed cases presented an improvement from *ρ* = 20% to *ρ =* 80%.

**Figure 8:**
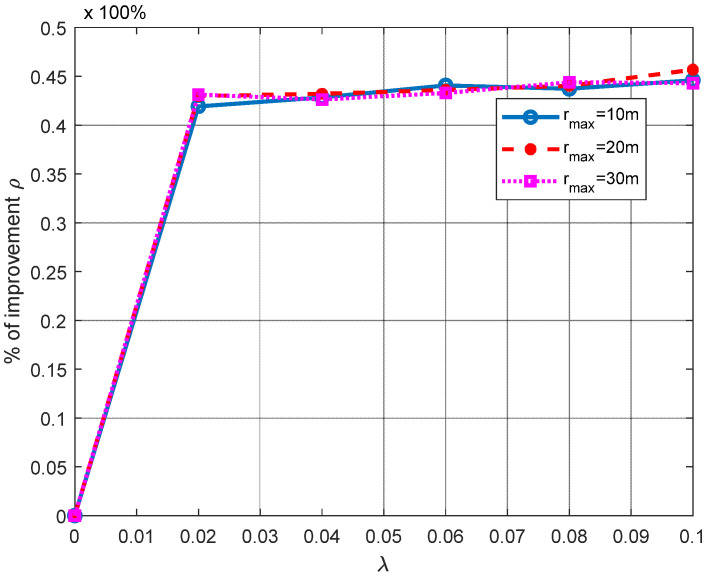
Performance improvement analysis of the RL algorithm for different *r*_max_.

In Figure 8, we analyze the RL performance based on the improvement percentage *ρ* using the mean RL in three ANs, for the scenario illustrated in Figure 1b, where three ANs are located at the corners of an equilateral triangle, *AN*_1_ (0,0,0), *AN*_2_ (*x*_2_ = *r*_max_,0,0) and *AN*_3_ (*x*_3_ = $r_{\max}/2$, *r*_max_/2,0), and *r*_max_ is increased gradually. In this figure, we can observe a very desirable behavior, as the RL performance is not degraded as the operational space is increased.

**Figure 9:**
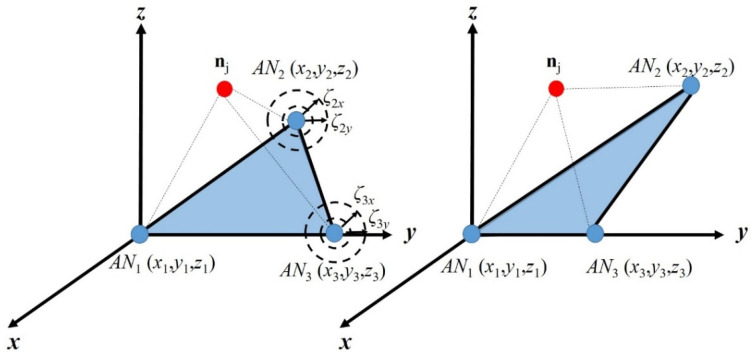
Generalization of the network scenario for evaluation.

Now, we evaluate the improvement *ρ* given by the RL algorithm when three ANs are available and for different locations of *AN*_2_ and *AN*_3_. Note that, by changing the position of these ANs defining the reference plane as that in Figure 9, we obtain all the possible shapes for the triangle on the reference plane. The evaluation network scenario is generalized by modifying the coordinates of *AN*_2_ and *AN*_3_, as illustrated in Figure 9.

**Figure 10:**
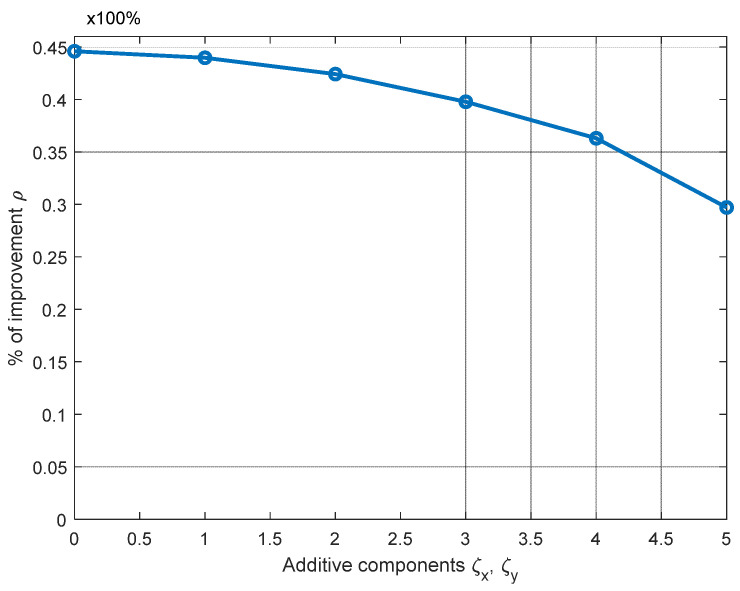
Improvement (*ρ*) evaluation of the RL algorithm for different locations of *AN*_2_ and *AN*_3_.

The deployment of *AN*_2_ is modified by adding to *x*_2_ and *y*_2_ random variables ±ζ_2*x*_ and ±ζ_2*y*_ respectively, while *z*_2_ is kept without alteration. The same modification is conducted for the coordinates of *AN*_3_. Let us recall that plane normalization by translation/rotation was carried out as explained before; therefore, we can consider that all the ANs are in *z* = 0. So, let us define the distances ζ*_ix_* and ζ*_iy_* (*i* = 1, 2, 3) as two uniformly distributed variables, defined in the ranges [0 ζ*_x_*] and [0 ζ*_y_*], respectively. (See axis-*x* in Figure 10.) This is performed in order to generalize the results given by the RL algorithm. In Figure 10, we can observe that, for increments below the 30% of *r*_max_ (ζ*_x_* = 1, ζ*_y_* = 1), the accuracy improvement *ρ* is not severely degraded, as *ρ* only decreases by approximately 11%.

**Figure 11:**
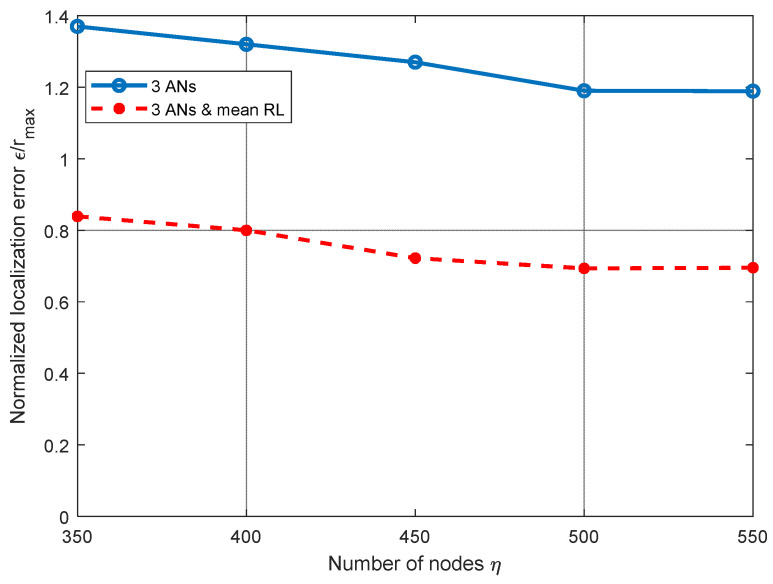
Normalized mean squared error *ε*/*r*_max_ vs. number of nodes *η* for multihop scenarios, *r*_max_ = 3 m.

For multihop scenarios, a direct link from the ANs to the node to locate may not be available. In Figure 11, we analyze the performance of the localization technique using three ANs as in Figure 1, over multihop scenarios in a defined cube with side *l* = *r*_max_. The shortest route from *AN_i_* to a node ***n****_j_*, is referred to as *R*_*i*,*j*_ and is obtained by Dijkstra’s algorithm, considering a homogenous reachability radius *r*_max_ = 3 m for all nodes in the network. For this analysis, the distance *d_ij_* from *AD_i_* to node ***n****_j_*, is approximated by the hop count in the route linking the ANs to ***n****_j_*. In Figure 11, we analyze the RL algorithm performance over a multihop route scenario for a different number of nodes *η* in the operational space. In this analysis, we can see that, as the number of nodes in the network grows, the distance error given by the single plane (three ANs) equations using the mean RL in three ANs tends to decrease. This can be explained by the fact that, when the number of nodes increases, the network connectivity improves and better routes may be obtained. Let us recall that, in this evaluation, distance estimation is based on the hop count method. Note that the improvement achieved by the RL algorithm is always above approximately 30%.

## 4. Conclusions

A recursive data fusion-based localization method suitable for 3D sensor networks is here proposed and its performance assessed both in line-of-sight scenarios (single hop) and in the presence of propagation obstructions (multihop environments) with three and four ANs. The 3D PL method is based on the space formulated by arbitrarily located ANs near the network operational scenario. The RL algorithm was formulated and it was observed that it achieves significant improvements under different scenarios. It was shown through simulations that different locations of ANs do not impair the performance of the RL algorithm. For multihop environments, it was also observed that, as the number of nodes increases, the performance of the PL 3D technique improves, as the estimated distances are improved by means of better (straight) routes.

## Figures and Tables

**Table 1:** Summary of localization methods.

Localization Method	Requirements	Accuracy
Classical trilateration with Kalman filter [6] (range-based).	RSSI estimations. At least 5% of nodes must be ANs, starting with 200 nodes.	Achieves an estimation error of around 100 feet in 1000 × 1000 × 100 feet-complex scenarios.
Multidimensional scaling (MDS)-based location techniques [7,10] (range-based).	Distance estimations, high node connectivity—10% of nodes must be ANs to achieve their best reported result.	Achieves an estimation error of around 0.12*R* in a 100*r* × 100*r* × 100*r* scenario with 10 anchor nodes.
Voronoi-based PL algorithm [11] (range-based).	RSSI estimations, high number of ANs.	Achieves an estimation error of around 0.12*r*_0_ for 8 ANs, for *r*_0_ = 8 m
Heuristic localization method [12] (free-range-based).	Hop count distance estimation, specific deployment of ANs. Four ANs required.	Achieves an error estimation of around 0.83*r*_max_ for a network with 600 nodes and 4 ANs.

## Data Availability

Not applicable.
